# Protocol for an interventional study to reduce postpartum weight retention in obese mothers using the internet of things and a mobile application: a randomized controlled trial (SpringMom)

**DOI:** 10.1186/s12884-021-03998-w

**Published:** 2021-08-23

**Authors:** Maki Kawasaki, Asako Mito, Masako Waguri, Yuichi Sato, Emiko Abe, Mayumi Shimada, Sayuri Fukuda, Yuki Sasaki, Kei Fujikawa, Takashi Sugiyama, Erika Ota, Jin Nakazawa, Tadashi Okoshi, Hidemi Takimoto, Yuka Honda, Eisuke Inoue, Yuji Hiramatsu, Naoko Arata

**Affiliations:** 1 Department of Health Policy, National Center for Child Health and Development, 2-10-1, OkuraTokyo, Setagaya 157-8535 Japan; 2 Division of Maternal Medicine, Center for Maternal-Fetal, Neonatal, and Reproductive Medicine, National Center for Child Health and Development, 2-10-1, OkuraTokyo, Setagaya 157-8535 Japan; 3 Department of Obstetric Medicine, Osaka Women’s and Children’s Hospital, Murodo-cho, Izumi, Osaka 840594-1101 Japan; 4 Obstetrics & Gynecology, TATE DEBARI Sato Hospital, 96, Wakamatsu-cho, Takasaki, Gunma 370-0836 Japan; 5 Department of Obstetrics and Gynecology, Ehime Prefectural Central Hospital, 83, Matusyama City, Ehime, Kasuga-cho 790-0024 Japan; 6 Link and Communication Inc, 4-1, Kioi-cho, Chiyoda, Tokyo, 102-0094 Japan; 7 Department of Obsterics and Gynecology, Ehime Uiversity Graduate School of Medicine, Touon City, Ehime, Sitsukawa 454791-0295 Japan; 8 Global Health Nursing, Graduate School of Nursing Sciences, St. Lukes International University, 10-1, Akashi-cho, Tyuuou, Tokyo, 1104-0044 Japan; 9 Faculty of Environment and Information Studies, Keio University, Fujisawa City, Kanagawa, Endo 5322252-0882 Japan; 10 Graduate School of Media and Governance, Fujisawa City, Kanagawa, Endo 5322252-0882 Japan; 11 National Institute of Health and Nutrition, 23-1, Toyama, Shinjuku, Tokyo, 162-8636 Japan; 12 Showa University Research Administration Center, Showa University, 1-5-8, Hatanodai, ShinagawaTokyo, 142-8555 Japan; 13 Okayama City General Medical Center, 3-20-1, KitanagaseomotemachiOkayama City, Okayama, 700-8557 Japan

**Keywords:** Obesity, Pregnancy, Weight, Internet of Things, Mobile phone

## Abstract

**Background:**

Obese pregnant women are known to experience poorer pregnancy outcomes and are at higher risk of postnatal arteriosclerosis. Hence, weight control during and after pregnancy is important for reducing these risks. The objective of our planned randomized controlled trial is to evaluate whether the rate of change in body weight in obese women before pregnancy to 12 months postpartum would be lower with the use of an intervention consisting of Internet of Things (IoT) devices and mobile applications during pregnancy to 1 year postpartum compared to a non-intervention group.

**Methods:**

Women will be recruited during outpatient maternity checkups at four perinatal care institutions in Japan. We will recruit women at less than 30 weeks of gestation with a pre-pregnancy body mass index ≥ 25 kg/m^2^. The women will be randomly assigned to an intervention or non-intervention group. The intervention will involve using data (weight, body composition, activity, sleep) measured with IoT devices (weight and body composition monitor, activity, and sleep tracker), meal records, and photographs acquired using a mobile application to automatically generate advice, alongside the use of a mobile application to provide articles and videos related to obesity and pregnancy. The primary outcome will be the ratio of change in body weight (%) from pre-pregnancy to 12 months postpartum compared to before pregnancy.

**Discussion:**

This study will examine whether behavioral changes occurring during pregnancy, a period that provides a good opportunity to reexamine one's habits, lead to lifestyle improvements during the busy postpartum period. We aim to determine whether a lifestyle intervention that is initiated during pregnancy can suppress weight gain during pregnancy and encourage weight loss after delivery.

**Trial registration:**

UMIN: UMIN (University hospital Medical Information Network) 000,041,460. Resisted on 18^th^ August 2020. https://upload.umin.ac.jp/cgi-open-bin/ctr_e/ctr_view.cgi?recptno=R000047278

**Supplementary Information:**

The online version contains supplementary material available at 10.1186/s12884-021-03998-w.

## Background

The increase in the number of overweight/obese individuals with a body mass index (BMI) ≥ 25 kg/m^2^ worldwide [1] has led to an increased proportion of overweight/obese pregnant women [2]. In Asia, because of poorer health prognoses, a BMI ≥ 23 kg/m^2^ is generally considered overweight [3], while in Japan, a BMI ≥ 25 kg/m^2^ is considered obese; therefore, pregnant women whose pre-pregnancy BMI is ≥ 25 kg/m^2^ are considered obese [4].

In general, obese pregnant women are known to have poorer pregnancy outcomes than non-obese pregnant women [5]. In Japan, obese pregnant women are at high risk of miscarriage, preterm birth, stillbirth, and congenital anomalies and are reportedly at an increased risk of conditions such as pregnancy-induced hypertension, gestational diabetes, preterm birth, large-for-gestational-age infants, excessively large infants weighing ≥ 4 kg, venous thrombosis, cesarean section, and postnatal bleeding associated with cesarean Sect. [6, 7].

Obese women may also develop metabolic syndrome, comprising diabetes, hypertension, and dyslipidemia, and are at a high risk of arteriosclerotic diseases such as heart disease and cerebrovascular accidents, resulting in long-term postnatal health problems [8, 9]. If gestational diabetes or pregnancy-induced hypertension occurs during pregnancy, these risks are further elevated [10–12].

Weight control during and after pregnancy in obese pregnant women is important for reducing the risk of negative pregnancy outcomes and to avoid the occurrence of diseases after pregnancy in the long-term. In particular, excessive weight gain during pregnancy is associated with a high risk of postpartum weight retention (PPWR)[13], as well as the risk of being obese or overweight in the long term after pregnancy[14]. However, few reports have detailed on the longitudinal changes in postpartum weight in Japanese and other East Asian populations.

In Europe and the United States, numerous randomized controlled trials have conducted lifestyle interventions during and after pregnancy for obese pregnant women. Lifestyle interventions during pregnancy that combine diet and exercise have been shown to suppress weight gain during pregnancy [15]. Postpartum lifestyle interventions have shown promise in reducing PPWR [16]; however, many of these studies involved relatively short postpartum intervention periods of approximately 12 weeks. Further, lifestyle interventions for obese women initiated during pregnancy and continued into the postpartum period could affect weight gain during and after pregnancy, although the number of studies implementing such interventions has been limited [17–19]. In addition, while interventions using the Internet of Things (IoT) such as online services and mobile phones have been shown to help suppress weight gain during pregnancy in obese women [20], no studies have reported on the effectiveness of IoT-based interventions performed during and after pregnancy in obese Asian women.

The objective of the present study is to evaluate whether the weight change in obese women at 12 months postpartum compared to before pregnancy will become lower with an intervention utilizing IoT devices and mobile applications during pregnancy to 1 year postpartum compared to without an intervention. This paper describes the design and methods of the planned SpringMom study.

## Methods/Design

This protocol follows the SPIRIT guidelines [21]. SpringMom will be a multicenter randomized controlled trial in which participants will be randomly allocated to an intervention at each facility. The intervention will last until 1 year postpartum, and the participants will be observed until 2 years postpartum.

**Fig. 1:**
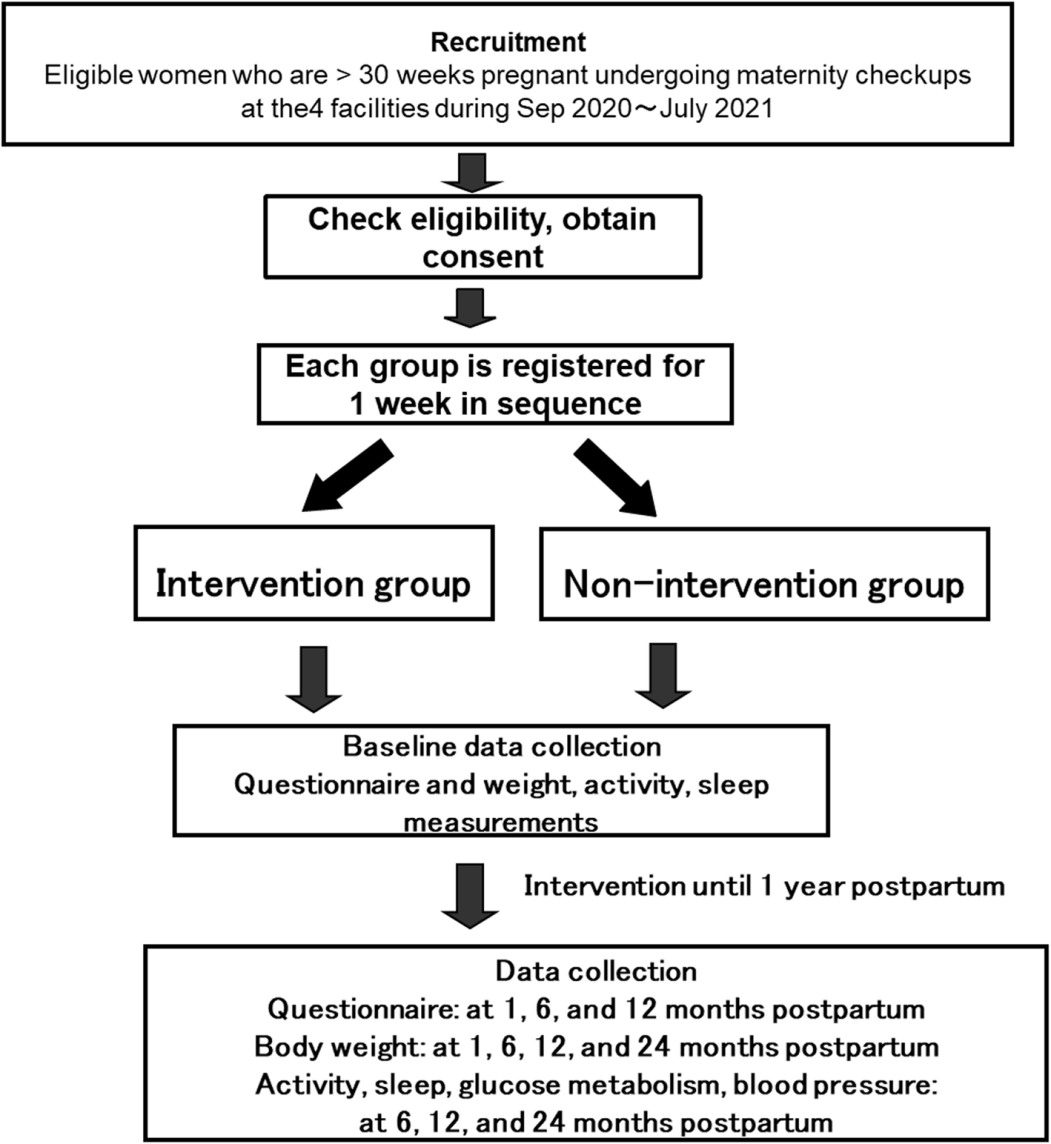
Flow chart of the study procedures

The enrollment period started in September 2020 and will end in June 2021, and the observation period started in September 2020 and will end in January 2024. Figure 1 shows the flow chart of the study procedures. This trial was approved by the ethics review board of the National Center for Child Health and Development, Tokyo, Japan.

### Trial setting

The trial will be conducted at four perinatal institutions (National Center for Child Health and Development (Tokyo), Osaka Women’s and Children’s Hospital (Izumi City), Sato Hospital (Takasaki City), and Ehime Prefectural Central Hospital (Matsuyama City)). Participants are being recruited the physician at each site while they attend outpatient maternity checkups, and their written informed consent is obtained.

### Participants

Pregnant women who are examined at the abovementioned four institutions will be eligible for the study.

The inclusion criteria are as follows:

1.  < 30 weeks' pregnant when consent is provided
2. Pre-pregnancy BMI ≥ 25 kg/m^2^

The exclusion criteria are as follows:

1.  < 20 years old and never married
2. Not a smartphone user or having a device with an operating system older than iOS 8.0 or Android 4.4
3. Diagnosed with diabetes before pregnancy or overt diabetes during pregnancy
4. Weighing ≥ 120 kg when consent is provided
5. Cannot communicate in Japanese
6. Deemed unsuitable for the trial by the physician in charge

### Allocation

Randomization will be conducted on the site level, not on the patient level. Every day during the enrollment period before starting recruitment, the trial secretariat will perform central allocation with a random number table to decide whether to enroll the participants in the intervention or non-intervention group at each site and, subsequently, will inform every site.

### Blinding

According to the nature of the intervention, the participants and research coordinator at each site will not be blinded to the allocation. However, the physician at each site will be blinded to the allocation.

### Intervention

The intervention will be initiated on the day consent is provided during pregnancy to 1 year postpartum. The IoT intervention for this trial is defined as (1) automatically generated advice based on data (weight, body composition, activity, sleep) measured using IoT devices and meal records and photographs recorded with a mobile application (Calo Mama Plus Pregnant woman Course®) and (2) provision of articles and videos related to obesity and pregnancy via a mobile application.

The following IoT devices will be distributed to all participants: body composition monitor and scale (HBF-227t, Omron Healthcare Inc., Tokyo) and fitness tracker (Fitbit Inspire®, Fitbit Inc., San Francisco, CA).

Participants in the intervention group will use a mobile application (Calo Mama Ninsanpu Plus®). The target weight 1 year postpartum has been set at 93% of the pre-pregnancy weight. Participants will monitor themselves daily to the extent possible using the IoT devices (weight and body composition monitor, activity, and sleep tracker) and by entering meal records and photographs into the mobile application.

The mobile application (Calo Mama Plus Ninsanpu Course®) has the following four functions:

1. It automatically generates personalized advice based on conditions during and after pregnancy. Personalized advice is generated automatically based on data (weight, body composition, activity, and sleep) measured with the IoT devices and meal records and photographs recorded by the mobile application, alongside considering how far long in the pregnancy the participant is or the number of weeks postpartum.
2. It provides articles based on the number of weeks of pregnancy or postpartum. The articles are delivered once or twice a week on themes that match the number of weeks of pregnancy or postpartum.
3. It provides breastfeeding advice. After delivery, advice on breastfeeding is provided that reflects the breastfeeding status (number of times the participant recorded breastfeeding and amount of milk).
4. It plots the body weight during pregnancy. Weight measurements during pregnancy are plotted on a graph and advice on weight changes based on the pre-pregnancy BMI is provided.

In addition, episodes from "Hana-chan's best chance for pregnancy and postpartum weight loss," a manga comic that discusses pregnancy and obesity, are automatically sent to the mobile application. The story consists of 10 episodes, with episodes 1–7 sent during pregnancy and episodes 8–10 sent after delivery (Supplementary Table 1).

The mobile application for pregnancy will be used during pregnancy until delivery; afterward, the participants will switch to a postpartum application, with the intervention resuming at 6 weeks postpartum and continuing until 1 year postpartum.

Participants in the non-intervention group will undergo regular maternity checkups, receive health guidance (usually until 1 month postpartum), and be provided with medical care covered by health insurance as needed. In Japan, women usually undergo maternity checkups approximately 15 times roughly throughout 10 months of pregnancy. The IoT devices (weight and body composition monitor, activity, and sleep tracker) must be worn when the data are collected (start of the study and at 1, 6, 12, and 24 months postpartum). The articles provided to the intervention group will also be available on a website.

During the study period, the research coordinator at each site will ask the participants in the intervention group if there is any inconvenience in using the device and mobile application. In both groups, useful information on infants will be regularly sent by e-mail after delivery to prevent dropouts.

**Fig. 2:**
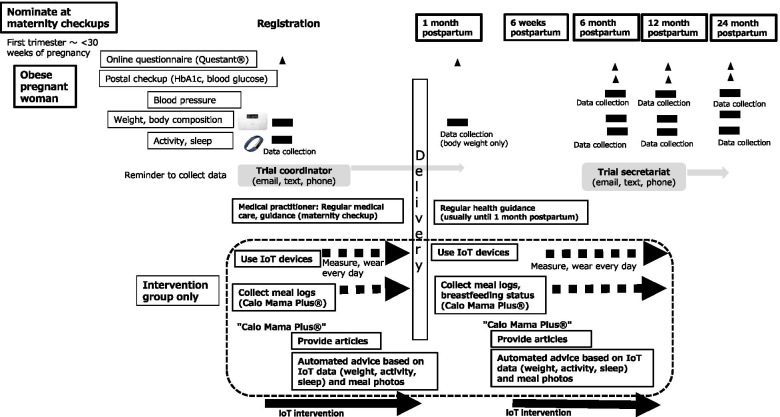
Study timeline for intervention and data collection

Figure 2 shows the study timeline for intervention and data collection.

If a participant withdraws her consent, cannot be contacted because of a move or hospital change, and the next pregnancy will continue for 14 weeks or more after delivery, participation will be discontinued. No criteria have been set for allocation modification.

### Data collection

All study participants must weigh themselves once at the start of the study and at 1, 6, 12, and 24 months postpartum and track their activity and sleep patterns for 1 week at the beginning of the study and at 6, 12, and 24 months postpartum. Body weight will be measured once a day in the morning using the weight and body composition monitor. Activity and sleep will be measured using an activity and sleep tracker, which will be worn on the non-dominant arm at all times, even during sleep, except during recharging (once every 5 days).

All participants will complete online questionnaires at the beginning of the study and at 1, 6, and 12 months postpartum. If any data are lacking, they will be collected from the medical records. Details of the questionnaires developed for this study are as follows (supplementary file):

1. Background and lifestyle (at the start of the study): items related to pregnancy and delivery history, medical history, family history, and exercise and dietary habits.
2. Edinburgh Postnatal Depression Score (EPDS) (start of study, 1 month postpartum) [22].
3. Breastfeeding status (intensity, duration) questionnaire (1, 6, 12 months postpartum).
4. Breastfeeding process questionnaire (1 month postpartum).
5. Behavioral changes (attitude, knowledge) (6, 12 months postpartum).
6. User-friendliness of devices, barriers to lifestyle improvements and behavioral changes, areas for improvement, etc. (6, 12 months postpartum) (both groups).
7. Desire to continue using the IoT devices after the trial ends (12 months postpartum) (intervention group only).

All participants will have blood samples taken at home via postal self-checkups at 6, 12, and 24 months postpartum to measure HbA1c, fasting glucose, total protein, albumin, AST, ALT, γ-GTP, total cholesterol, HDL cholesterol, triglycerides, urea nitrogen, creatinine, and uric acid. To collect blood samples, the trial secretariat will send test kits to the participants for postal self-checkups. Results of the postal self-checkups will be returned by mail to the participants within 6 weeks.

All participants will measure their blood pressure at home over 3 days at 6, 12, and 24 months postpartum. The HBF-227t sphygmomanometer manufactured by OMRON Healthcare Co. will be used. Measurements will be taken twice a day in the morning (within 1 h of waking up, after urinating, before taking any medication, and after resting for 1–2 min) and before going to sleep (after resting in the sitting position for 1–2 min). In principle, two measurements will be taken each time.

Table 1 shows the schedule for the trial.

**Table 1:** Study timeline for intervention and data collection

		Trial duration								
		Obtain consent		Intervention						Completion
Pregnancy, postpartum			Start of trial	During pregnancy	Delivery	1 month postpartum	6 weeks postpartum	6 months postpartum	12 months postpartum	24 months postpartum
	Obtain consent	×								
	Registration	×								
Regular management	Regular medical care, guidance (maternity checkups)Regular health guidance			○ (maternity checkup→→		○ (1-month postpartum checkup)				
Measure at home	Weight, body composition		○			○		○	○	○
	Activity, sleep		○					○	○	○
Blood pressure	Blood Pressure							○	○	○
Postal checkups	Postal checkup (blood glucose, HbA1c, etc.)							○	○	○
App intervention	Weight, activity, sleep		●	→→→→→→→			●→→→	→→→→→→	→	
	Meal photos		●	→→→→→→→			●→→→	→→→→→→	→	
Mother and Child Handbook	Maternity checkup data (urine test, blood pressure, weight)					○				
	Pregnancy outcome data					○				
Questionnaire	Background, lifestyle		○							
	EPDS		○			○				
	Breastfeeding status					○		○	○	
	Breastfeeding process					○				
	Behavioral changes (attitude, knowledge)		○					○	○	
	User-friendliness of devices							○	○	
	Desire to continue using IoT after end of study								●	

○ Both groups, ● intervention group only

If there are missing data, the research coordinator at each site and trial secretariat will contact the participants. Data from the weight and body composition monitor, activity and sleep tracker, and online questionnaires will be stored on the Internet server. The data file will be secured with a password, and access to information will be limited to designated members such as the researchers and the trial secretariat. Paper-based documents such as consent forms will be stored at each site.

### Primary outcome

The primary outcome will be the ratio of change in body weight (%) from pre-pregnancy to 12 months postpartum.

The pre-pregnancy weight will be self-reported, as weights reported by obese individuals have been reported to differ only slightly from actual measurements. The body weight at 12 months postpartum will be directly retrieved from the smartphone application as measured by the device.

### Secondary outcomes

The following items will be evaluated (shown in Table 2):

**Table 2:** Primary and secondary outcomes

Outcomes				Trial duration							
					Intervention						Finished
				Registration	During pregnancy	Delivery	1 montha postpartum	6 weeks postpartum	6 months postpartum	12 months postpartum	24 months postpartum
Primary			Rate of change in body weight from before pregnancy to 12 months postpartum	×						×	
Secondary	Health outcomes	Bothgroup	Pregnancy outcomes (cesarean section rate, preterm birth rate, LGA rate, excessively large baby rate, pregnancy-induced hypertension rate, gestational diabetes rate, etc.)			×					
			Rate of change in body weight from before pregnancy	×			×		×		×
			Percentage who reached the weight target						×	×	×
			Body fat ratio				×		×	×	×
			Hypertension rate						×	×	×
			Impaired glucose tolerance rate						×	×	×
			Edinburgh Postnatal Depression Score ≥9 rate	×			×				
	Behavioral changes		Number of steps	×					×	×	×
			Sleep	×					×	×	×
			Attitude, knowledge	×					×	×	
			Breastfeeding rate				×		×	×	
			Breastfeeding process				×				
		Intervention group only	Percentage of carbohydrates, fat, protein, dietary fiber in diet	×					×	×	
	Use of IoT/mobile app		Rate of use of weight-body composition monitor, activity-sleep tracker	×					×	×	
			Number of logins to mobile app, browsing time	×					×	×	
			Meal image/content upload status	×					×	×	
			Intervention program continuation rate						×	×	
			Adherence	×					×	×	×
			Desire to continue using IoT after end of study	×						×	
		Bothgroup	User-friendliness of the devices	×					×	×	

#### Health outcomes

- ✓ Pregnancy outcomes (gestational age at delivery, cesarean section rate, preterm birth rate, excessively large baby rate, birth height, birth weight, SD value, large-for-gestational-age rate, pregnancy-induced hypertension rate, gestational diabetes rate, etc.)
  Maternity checkup and pregnancy outcome data will be collected from the Maternal and Child Health Handbook.
- ✓ Weight gain during pregnancy (kg)
  Calculated as: (weight immediately before delivery retrieved from the Maternal and Child Health Handbook)—(pre-pregnancy weight).
- ✓ Change in weight from before pregnancy to 1, 6, 12, and 24 months postpartum (kg)
- ✓ Rate of change in weight from before pregnancy to 1, 12, and 24 months postpartum (%)

The postpartum weights will be directly retrieved from the smartphone application as measured by the device.

- ✓ Percentage of participants who will reach the target weight (at 6, 12, and 24 months postpartum) (%)
  A participant will be considered to have reached the postpartum target weight if/when their body weight declines to 93% of the weight before pregnancy.
- ✓ Change in body fat ratio from 1 month postpartum to 6, 12, and 24 months postpartum
- ✓ Percentage of participants with hypertension (excluding those having been diagnosed before pregnancy)
  The mean of the two blood pressure readings taken at home in the morning and before going to bed will be used. If only one blood pressure reading is taken, that value will be used. Hypertension is defined as a 3-day mean of ≥ 135 mmHg and/or ≥ 85 mmHg.
- ✓ Percentage of participants with impaired glucose tolerance or diabetes (at 6, 12, and 24 months postpartum)
  Postpartum impaired glucose tolerance is defined as fasting blood glucose ≥ 110 mg/dl and ≤ 125 mg/dl or HbA1c ≥ 6.0% and ≤ 6.4% in the at-home blood samples. Diabetes is defined as a fasting blood glucose level of ≥ 126 mg/dl or HbA1c is ≥ 6.5%.
- ✓ Percentage of participants with EPDS ≥ 9 (start of the study and at 1 month postpartum)

#### Behavioral changes

- ✓ Attitude, knowledge
  (Based on relevant questionnaire items).
- ✓ Mean total caloric intake (kcal/day), percentage of carbohydrates in total caloric intake (%)
- ✓ Mean carbohydrate intake (g/day)
- ✓ Percentage of fat in total caloric intake (%)
- ✓ Mean fat intake (g/day)
- ✓ Percentage of protein in total caloric intake (%)
- ✓ Mean protein intake (g/day)
- ✓ Mean dietary fiber intake (g/day)
- ✓ Mean salt intake (g/day)
  For the intervention group only, the above will be calculated from the data entered into the mobile application (Calo Mama Plus Ninsanpu Course®).
- ✓ Number of steps
- ✓ Sleep
- Data from the activity and sleep monitor will be used
- ✓ Breastfeeding rate (at 1 and 6 months postpartum)

(Based on relevant questionnaire items)

- ✓ Duration of breastfeeding (12 months postpartum)
  (Based on relevant questionnaire items)
- ✓ Indicators of breastfeeding process, breastfeeding desires (at 1 month postpartum)
  (Based on relevant questionnaire items)

#### Use of IoT devices/mobile applications (*intervention group only)

- ✓ Rates of IoT device wearing, measurements (activity tracker, weight, and body composition monitor)
- ✓ Mean logins per day to mobile applications, hours viewed per day
- ✓ Meal image/content upload status
- ✓ Breastfeeding video viewing status
- ✓ Intervention program continuation rate

Dropping out of the intervention program is defined as follows: If measurement data are not registered for 2 weeks or longer, an investigator will contact the participant by e-mail or phone to remind them to register their data. If they cannot be contacted or do not register data, contact will be attempted again every 2 weeks. If the participant still cannot be contacted at 6 or 12 months postpartum, or if they do not register any data, they will be removed from the program.

- ✓ Adherence
  Low adherence is defined as bottom 20% use of the IoT devices (weight, number of steps, etc.), of opening the mobile applications or of inputting meals during a 2-week period at the beginning of the study and at 6 and 12 months postpartum.
- ✓ User-friendliness of the devices
  (Based on relevant questionnaire items)
- ✓ Desire to continue using the IoT devices after the trial ends

(Based on relevant questionnaire items).

### Sample size

Assuming a -2% weight change at 12 months postpartum compared to before pregnancy in the non-intervention group, the trial was designed to detect whether the IoT/mobile application intervention would result in a -5% weight change at 12 months postpartum. To conduct a two-sided test with a standard deviation of 8% and significance level of 5% at 80% power, 226 participants in both groups are needed. Assuming loss of approximately 20% of participants from exclusion or drop-out, the target number of registrations is set to 270 women.

There are 6,000 deliveries each year at the trial institutions. If 10% of the women are obese, the total number comes to 600 per year. As pregnant women at up to 30 weeks of gestation are eligible for registration, 250 pregnant women at up to 30 weeks of gestation who have already visited the outpatient maternity clinic for checkups and 250 pregnant women who newly visit during the 5-month registration period will be eligible. Hence, it is expected to be possible to register the target number of participants.

### Statistical methods

The full analysis set (FAS) will comprise the randomized participants, excluding those who meet any of the following conditions:

-
  - Trial intervention not implemented
-
  - Endpoints not measured at baseline and after the start of the intervention
-
  - Did not meet the inclusion criteria after enrollment or randomization
  The per protocol set will comprise a subset of the FAS that will follow the trial protocol, excluding those who fail to meet any of the following conditions:
-
  - Scale: Data obtained from at least two measurements at 1, 6, and 12 months postpartum (1 day)
-
  - Fitbit: Measurements taken at both 6 and 12 months postpartum (7 days)
-
  - Application meal entries: At least one entry during 2 weeks at 6 and 12 months postpartum
-
  - Obesity articles: Viewed 8 of the 10 articles

### Analysis of primary outcome

Change in weight at each measurement point will be analyzed by fitting a mixed-effects model for repeated measures. The explanatory variables of the model include the pre-pregnancy body weight, measurement time points, intervention group, and interaction between measurement time points and intervention group. The variance structure is unstructured (variance at each time point is heterogenous). Using this model, the least mean square of the rate of change in body weight at 12 months postpartum and its 95% confidence interval are estimated for each group, and differences between the groups are compared using t-tests (two-sided). The significance level of the test is set at 0.05.

### Analysis of secondary outcomes

For continuous variables, the mean and 95% confidence interval will be calculated for each group and t-tests will be used for group comparisons. For discrete variables, the number, proportion, and 95% confidence interval for the proportion will be calculated for each group, and the chi-square test will be used for group comparisons.

Regarding the breastfeeding proportion, ≥ 80% breastfeeding (daily milk amount ≤ 125 ml at 1 month postpartum and ≤ 164 ml at 6 months postpartum) will be used for between-group comparisons. Regarding the dietary items, the means and 95% confidence intervals will be calculated for each item at each time point (at the start of the study and 6 and 12 months postpartum) in the intervention group. The same analysis will be performed to analyze changes from the start of the study to 6 and 12 months postpartum.

For device usage, the use of activity tracker meters, wearing the weight/body composition monitor, and performance of measurements with the weight/body composition monitor at each time point (6 and 12 months postpartum) will be summarized and compared between the groups. In the group that will not use the devices at the beginning of the study, the number, proportion, and 95% confidence interval for the proportion will be calculated for participants who started using the devices at 6 and 12 months postpartum.

Regarding mobile application use, the mean and 95% confidence intervals will be calculated for the number of logins and viewing time at each time point in the intervention group (at 6 and 12 months postpartum). For uploading of meal images (at 6 and 12 months postpartum) and viewing of breastfeeding videos (during pregnancy and at 6 weeks postpartum), the number, proportion, and 95% confidence interval will be calculated for the intervention group.

Regarding the continuation of the intervention program (at 6 and 12 months postpartum), the number, proportion, and 95% confidence interval will be calculated for the intervention group.

Regarding adherence, the number, proportion, and 95% confidence interval for participants who enter data via the IoT devices and mobile applications ≥ 80% of the time during 2 weeks at each time point (at the beginning of the study and 6 and 12 months postpartum) will be calculated for the intervention group (using data from a data integration platform). In addition, the number, proportion, and 95% confidence interval of participants who will open the application and enter meals into the application for ≥ 4 days per week will be calculated for the intervention group.

### Subgroup analysis

The following subgroup analyses will be performed on the primary and secondary endpoints (pregnancy outcomes, body weight, body fat, blood pressure, impaired glucose tolerance, EPDS, diet, health, and breastfeeding):

• Among those who will perform ≥ 80% or < 80% of measurements of body weight, body composition, and activity throughout the study period
• Among those who will perform ≥ 80% or < 80% of measurements of body weight and body composition throughout the study period
• Among those who will use the activity tracker for ≥ 80% or < 80% of the study period
• Among those who will perform ≥ 60% or < 60% of measurements of body weight, body composition, and activity throughout the study period
• Among those who will perform ≥ 60% or < 60% of measurements of body weight and body composition throughout the study period
• Among those who will use the activity tracker for ≥ 60% or < 60% of the study period
• Among those with body weight at registration ≥ / < the median
• Among those with body composition measurement at registration ≥ / < the median
• Among those with activity tracker measurement at registration ≥ / < the median

### Confidentiality

All data will be anonymized using a research ID number. Personal data including name, e-mail, telephone number, and address contained in the consent forms of participants will be stored at each site and by the trial secretariat. The research ID and corresponding table will be stored at each site.

### Monitoring

Although this will be an intervention trial, the intervention is not considered to be invasive. Thus, no data monitoring committee or auditing will be needed. The research coordinator at each site and trial secretariat will regularly discuss the progress of the study and any issues to be addressed. Any modification or inquiry regarding the study will be communicated through the trial secretariat.

### Harms

The intervention planned for this study is not considered to be harmful to the participants. If adverse events occur, the trial secretariat will take appropriate measures in cooperation with the principal investigator at each site.

### Dissemination policy

The study findings will be disseminated at peer reviewed scientific journals and presented at international conferences and conferences in Japan and other countries.

## Discussion

This paper describes the protocol for a randomized controlled trial that aims to determine whether a lifestyle intervention using IoT devices and mobile applications performed during pregnancy to 1 year postpartum will affect weight loss in obese women at 1 year postpartum.

In Japan, the proportion of women of childbearing age with BMI ≥ 25 kg/m^2^ is approximately 10% (National Health and Nutrition Survey, Ministry of Health, Labour, and Welfare). These women tend to have poorer pregnancy outcomes [6, 7]. It has also been reported that in women with gestational diabetes, the risk for developing diabetes mellitus over a mean of 2.2 years postpartum was 3.2 among those who were obese before becoming pregnant compared to non-obese women [23]. However, other long-term data concerning postnatal outcomes are unavailable. In addition, there have been few reports on longitudinal weight changes from before to after pregnancy in obese East Asian women, including Japanese, and no reports on interventions using IoT devices during and after pregnancy in obese Asian women.

The significance of starting the lifestyle intervention during pregnancy in the present study is as follows: (1) lifestyle interventions that start during pregnancy can suppress weight gain during pregnancy, leading to greater postpartum weight loss; (2) as pregnancy is a good opportunity to reexamine one's lifestyle habits, behavioral changes made during pregnancy could lead to lifestyle improvements during the busy postpartum period; and (3) it has been suggested that providing guidance concerning breastfeeding during pregnancy can increase the breastfeeding rate. Once established, a woman could expend 500 kcal per day by breastfeeding, which could help her lose weight. However, obesity is reportedly associated with shorter periods of breastfeeding [24]. In addition, the postpartum period, especially at approximately 6 months postpartum when the baby starts weaning and breast milk decreases, can be a time when weight tends to rebound. Hence, intervening at this time is likely to be important for obese women. Thus, if obese women receive suitable advice at the appropriate time concerning suitable lifestyle habits and breastfeeding is promoted, pregnancy and the first year postpartum period could be a great opportunity for weight correction. However, currently, there are no systems or studies that support this.

In the present study, we will use manga comics as a tool for educating obese pregnant women. Manga comics are commonly employed in Japanese educational culture. In Japan, a wide variety of manga characters appear in textbooks used in compulsory education. Some advantages of manga comics are that (1) use of friendly characters and expressions can increase interest and motivation for learning, (2) use of easy-to-understand illustrations can make it easier to convey content that may be difficult to express in writing, (3) the story can be tailored so the context, background, and content are easy to grasp; (4) placing teachers and students in the manga tends to place the reader in the student's position and help them participate in the process of learning through dialogue with the teacher [25]. To increase awareness of obesity, pregnancy, and childbirth, we created a manga comic that follows a main character (Hana-chan) with a pre-pregnancy BMI of 30 kg/m^2^ from when she becomes pregnant through childbirth and the postpartum period. The comic will be sent to the participants' smartphones.

(Hana-chan application website: https://www.ncchd.go.jp/hospital/about/section/perinatal/bosei/bmi.html).

In the present study, a mobile application will automatically provide individualized advice based on the participant's status during and after pregnancy. This personalized automated advice will consider the weeks of pregnancy or the postpartum period and will be based on the weight, activity, and sleep measurements acquired with the IoT devices and meal photographs and records uploaded to the mobile application. Lifestyle-related interventions such as coaching provided over mobile platforms have been found to have the same effects on weight loss as in-person interventions for obese non-pregnant women [26]. Hence, personalized advice is expected to be effective.

Smartphones and other eHealth technologies are becoming more common in today's society. In Japan, more than 90% of individuals in their 20 s and 30 s use smartphones [27], according to a survey on IT media use and information behavior in 2019 (ICT Policy Institute, Ministry of Internal Affairs and Communications, September 2020). Providing advice through articles and other means delivered via smartphone and receiving automated advice based on IoT device measurements or data recorded with a smartphone application may help promote behavioral changes that will result in sustainable weight loss in obese women. If this can be achieved, interventions consisting of IoT devices and mobile applications could be used in actual clinical practice.

## Supplementary Information

**Additional file 1: Supplementary Table S1**. Hana-chan's best chance for pregnancy and postpartum weight loss.

**Additional file 2: **Questionnaires.

## Data Availability

The datasets generated and/or analyzed during the current study are available from the corresponding author on reasonable request.

## Abbreviations

BMI: Body mass index
PPWR: Postpartum weight retention
IoT: Internet of Things
