# Supplementary material for: Protocol for an interventional study to reduce postpartum weight retention in obese mothers using the internet of things and a mobile application: a randomized controlled trial (SpringMom)
Source: BMC Pregnancy Childbirth. 2021 Aug 23;21:582. doi: 10.1186/s12884-021-03998-w (PMC8381573; doi:10.1186/s12884-021-03998-w)
Supplement: Supplementary file 1 — Additional file 1: Supplementary Table S1. Hana-chan's best chance for pregnancy and postpartum weight loss. [file 12884_2021_3998_MOESM1_ESM.docx]

Supplementary Table 1

|  | Title | Outline |
| --- | --- | --- |
| Episode 1 | Story of Hana-chan's great chance to lose weight during and after pregnancy | Detailing the relationship between obesity and pregnancy outcomes and diseases after pregnancy over the long term |
| Episode 2 | Pregnancy is a great opportunity for weight management | Recommends losing 3–7% of one's body weight compared to the pre-pregnancy weight 1 year after delivery |
| Episode 3 | Proper diet | Explains (1) a balanced diet (2) < 6.5 g/day of salt intake, (3) a low GI diet |
| Episode 4 | Proper exercise | Encourages moderate intensity exercise for 20–30 minutes daily |
| Episode 5 | Gestational diabetes | Explains the risk factors and treatments for gestational diabetes |
| Episode 6 | Pregnancy-induced hypertension | Explains the risk factors for pregnancy-induced hypertension and how to reduce salt intake |
| Episode 7 | Breastfeeding | Explains the benefits of breastfeeding for mothers and children.  Six videos with a 3-minute duration that promote breastfeeding |
| Episode 8 | Sleep, life rhythms, dealing with stress | Describes where to get help in times of stress |
| Episode 9 | Postpartum life | Explains key times for postpartum weight loss |
| Episode 10 | Looking to the next pregnancy | Discusses the timing of the next pregnancy and the effects of weight loss on pregnancy outcomes |
