# Supplementary material for: Protocol for an interventional study to reduce postpartum weight retention in obese mothers using the internet of things and a mobile application: a randomized controlled trial (SpringMom)
Source: BMC Pregnancy Childbirth. 2021 Aug 23;21:582. doi: 10.1186/s12884-021-03998-w (PMC8381573; doi:10.1186/s12884-021-03998-w)
Supplement: Supplementary file 2 — Additional file 2: Questionnaires. [file 12884_2021_3998_MOESM2_ESM.docx]

The questionnaire

**Background and lifestyle (at the start of the study)**

1. 1. Have you been told the following illnesses during this pregnancy?

1-1. Gestational diabetes

□ Yes □ No

1-2. Preeclampsia

□ Yes □ No

2. Have you been told the following illnesses before this pregnancy?

2-1. High blood pressure (taking blood pressure medication or had a hospital blood pressure of 140/90 mmHg or higher)

□ Yes □ No

2-2. Thyroid disease

□ Yes □ No

2-3. Collagen disease, antiphospholipid antibody syndrome

□ Yes □ No

2-4. Chronic kidney disease

□ Yes□ No

2-5. Others ()

2. Please tell us if you know the blood pressure before this pregnancy

□ /□ mmHg

3. 3. Do you have any of the following illnesses in your family?

3-1 Diabetes

□ Yes (father, mother, brothers and sisters) □ No

3-2 Hypertension

□ Yes (father, mother, brothers and sisters) □ No

3-3 Angina, myocardial infarction, aortic aneurysm

□ Yes (father, mother, brothers and sisters) □ No

3-4 Stroke

□ Yes (father, mother, brothers and sisters) □ No

4. Please tell us your weight when you were 20 years old. ‥

□□□ .□ kg

5. Please tell us the heaviest weight ever except during pregnancy and the age at that time.

□ /□ mmHg、□ years old

6. Please tell us your pre-pregnancy weight.

□□□ .□ kg

7. Please tell us the minimum weight after pregnancy.

□□□ .□ kg

8. Please enter your height.

□□□. □ cm

9. Please enter the weight that you think is best at the time of delivery.

□□□ .□ kg

10. Do you drink ?

(1) No drinking habit (2) Stopped before pregnancy　(3) Stop after finding out that you are pregnant (4) I'm still drinking

11. Do you smoke ?

(1) No smoking habit (2) Stopped before pregnancy (3) Stop after finding out that you are pregnant　 (4) I'm still smoking

12. Do you eats too much?

□ Yes □ No

13. How fast do you eat?

□ Quite fast □ Slightly fast □ Normal □ Slightly slow □ Quite slow

14. How often do you eat "breakfast"?

□ Less than once a month □ 1-3 times a month □ 1-2 times a week □ 3-4 times a week □ 5-6 times a week □ Eat daily

15. How often do you "eat out"?

□ Less than once a month □ 1-3 times a month □ 1-2 times a week □ 3-4 times a week □ 5-6 times a week □ Eat daily

16. The following conditions are not recommended for exercise during pregnancy. Please answer.

□ Have a heart or lung disease

□ Imminent miscarriage or an imminent premature birth.

□ Short cervical canal or cervical asthenia

□ Experienced or ruptured water less than 37 weeks pregnant

□ Having genital bleeding

□ Placenta previa or low placenta.

□ High blood pressure (Upper blood pressure is 140 mmHg or higher or lower blood pressure is 90 mmHg or higher)

□ Orthopedic problems such as arthritis

□ Severe anemia

□ My doctor have not premitted to exercise

□ I don't feel like exercising

17. Have you ever been on a diet?

□ Yes □ No

To those who answered yes. How many times have you been on a diet?

(1).1 to 5 times (2).6 to 10 times (3).11 to 15 times (4). No more

**Breastfeeding status questionnaire (1, 6, 12 months postpartum)**

1.Please tell us current lactation intensity **(1, 6 months postpartum)**

(1) breast milk only

total number of feedings per day □times/ per day

(2) breast milk and formula feeding

(a) total number of breast milk □times/ per day

(b) total number of formula feeding □times/ per day

(c) quantity of formula per feeding □ml

2. Are you currently breastfeeding your baby? (**12 months postpartum)**

1) I'm giving

2) I gave it before, but I haven't given it now.

→ When I quit, around the month after birth □month

3) I have never given it

3. Did you have your next pregnancy? (**12 months postpartum)**

1) No.

2)Yes: Last menstrual date

**Breastfeeding process questionnaire (1 month postpartum)**

1. Did you tell the staff at the birthing facility that you want to breastfeed?

□ Yes □ No

2. Immediately after giving birth * Did you do Skin to Skin?

* Skin to Skin = Sticking mother's skin to baby's skin

□ Yes □ No

3. Did you breastfeed within 1 hour of giving birth?

□ Yes □ No

4. Did the hospital staff give you breastfeeding instructions while you were in the hospital?

□ Yes □ No

5. Did you formula feeding from the hospital when you were discharged?

□ Yes □ No

6. Did you go to the breastfeeding clinic after leaving the hospital? (Including guidance from a midwife at home)

□ Yes □ No

7. Did you breastfeed more than 7 times a days for the first month after giving birth?

□ Yes □ No

**Behavioral changes (attitude, knowledge) (6, 12 months postpartum)**

1.Please tell us about the current levels of 6 items below.

(1) Calorie adjustment

□ I'm not interested

□ I want to improve it, but I can't do it

□ A little can be done about the goal

□ You can roughly execute the goal

□ Almost achieved / or continued

(2) Nutritional balance

□ I'm not interested

□ I want to improve it, but I can't do it

□ A little can be done about the goal

□ You can roughly execute the goal

□ Almost achieved / or continued

(3) Snack

□ I'm not interested

□ I want to improve it, but I can't do it

□ A little can be done about the goal

□ You can roughly execute the goal

□ Almost achieved / or continued

(4) Eating out / drinking

□ I'm not interested

□ I want to improve it, but I can't do it

□ A little can be done about the goal

□ You can roughly execute the goal

□ Almost achieved / or continued

(5) Exercise

□ I'm not interested

□ I want to improve it, but I can't do it

□ A little can be done about the goal

□ You can roughly execute the goal

□ Almost achieved / or continued

(6) Sleep

□ I'm not interested

□ I want to improve it, but I can't do it

□ A little can be done about the goal

□ You can roughly execute the goal

□ Almost achieved / or continued

2. Do you know that your metabolism will improve if you lose 3% to 7% of your body weight?□ Applicable

□ Somewhat applicable

□ Somewhat not applicable

□ Not applicable

3. Do you know that breastfeeding improves post partum weight retention?

□ Applicable

□ Somewhat applicable

□ Somewhat not applicable

□ Not applicable

4. Do you know that sleep and weight are related?

□ Applicable

□ Somewhat applicable

□ Somewhat not applicable

□ Not applicable

5. Do you that it is important to eat a diet with proper energy and nutritional balance?

□ Applicable

□ Somewhat applicable

□ Somewhat not applicable

□ Not applicable

6. Do you know that the intensity of exercise should be set to an intensity that you　feel a little tight but allows you to continue talking to others?

□ Applicable

□ Somewhat applicable

□ Somewhat not applicable

□ Not applicable

**User-friendliness of devices, barriers to lifestyle improvements and behavioral changes, areas for improvement, etc. (6, 12 months postpartum)**

1) Did you have any problems with the IoT device?

□ It was troublesome to use multiple apps

□ It was troublesome to transfer weight and blood pressure data

□ I was reluctant to send meal images on my smartphone (intervention group only)

□ Charging Fitbit® was troublesome

□ It was difficult to understand how to use the app

□ There was no particular problem

2) Please tell us if there are any obstacles to improving your lifestyle.

□ Unconfident

□ I can't take time for childcare or work

□ There is no understanding or support from the surroundings

□ free description ( )

3) How do you think it can be improved?

□ Regular hospital visits

□ Regular health guidance

□ Support in the app

□ Group efforts with people who had the same goals as me

□ Content that the family could work on together.

□ free description ( )

**Desire to continue using IoT after trial ends (12 months postpartum)**

1) Did you have a medical examination regarding glucose tolerance, blood pressure and weight after giving birth? (Both groups)

□ None

□ 1-2 times

□ 3 times-5 times

□ 6 times or more

2) Please tell us about the changes in your dietary consciousness that occurred by participating in this study (multiple choices allowed).

(Both groups)

□ A change in what you eat during snacks

□ Reduced carbohydrates when eating

□ Started to reduce fat when eating

□ Started to worry about how to eat without raising blood sugar level

□ Started to worry about the amount of salt

□ Started to eat regularly in the morning, day and night

□ Interested in low-carbohydrate convenience stores.

□ I stopped drinking juice

□ The type of liquor has changed

□ I haven't realized the change yet

3) Please tell us about the behavioral changes that have occurred to you by participating in this study (multiple choices allowed).

(Both groups)

□ Being aware of the number of steps on the activity meter

□ I got into the habit of walking consciously every day

□ I became to use the stairs

□ I became aware of sleep

□ I haven't realized the change yet

4) Please tell us about the changes in your feelings that happened to you by participating in this study (multiple choices allowed).

(Both groups)

□ I wanted to know more because I became more aware of my health.

□ It's fun to lose weight

□ I feel better and feel more positive.

□ I became to think about the health of my family

□ I haven't realized the change yet

5) Please tell us about changes in your partner's consciousness and behavior by participating in this study (multiple choices allowed).

(Both groups)

□ Increased awareness of health

□ Improved eating habits of partners

□ The amount of activity of your partner has increased

□ The obesity of your partner was improved (only if there was obesity)

□ The life rhythm of your partner has been improved

□ I don't feel the change

□·I do not want to answer

6) Please answer about the contents of the smartphone column and smartphone automatic advice. (Intervention group only)

6-1) Please select the desired one regarding the number of smartphone columns.

□ The frequency of delivery was low

□ The frequency of delivery was satisfactory

□ The frequency of delivery was high

6-2) Please select the desired content for your smartphone column.

□ It was very helpful

□ It was a little helpful

□ I can't say either

□ It was not very useful

□ It didn't help at all

6-3) Please choose the one you want regarding the frequency of automatic smartphone advice.

□ The frequency of delivery was low

□ The frequency of delivery was satisfactory

□ The frequency of delivery was high

6-4) Please choose the one you want for automatic smartphone advice.

□ It was very helpful

□ It was a little helpful

□ I can't say either

□ It was not very useful

□ It didn't help at all

7) Do you want to continue using this service even after the program ends? (Intervention group only)

□ I want to use it if it is free

□ I want to use it for a fee (less than 500 yen per month)

□ I want to use it for a fee (about 500 yen to 1000 yen every month)

□ I want to use it for a fee (about 1000 yen to 2000 yen every month)

□ I want to use it for a fee (about 2000 yen to 3000 yen every month)

□ I want to use it for a fee (3000 yen or more every month)

□ I do not want to continue using it

8) Please indicate your satisfaction with each item of this program. (Intervention group only)

8-1) Overall satisfaction

8-2) Smartphone column

8-3) Smartphone automatic advice

8-4) Easy to understand service

□ Very satisfied

□ Slightly satisfied

□ I can't say either

□ Slightly dissatisfied

□ Extremely dissatisfied

9) Please select all that apply to the reason why you were able to continue this program ((multiple selections allowed) (intervention group only)

□ Because the content of the advice was reliable

□ Because the app was easy to use

□ Because the effect of weight loss came out

□ Because my family supported me

□ There is no particular reason to think of it
